# Comprehensive miRNA and DNA Microarray Analyses Reveal the Response of Hepatic miR-203 and Its Target Gene to Protein Malnutrition in Rats

**DOI:** 10.3390/genes13010075

**Published:** 2021-12-28

**Authors:** Kaoru Takahashi, Huijuan Jia, Shoko Takahashi, Hisanori Kato

**Affiliations:** Health Nutrition, Graduate School of Agricultural and Life Sciences, The University of Tokyo, 113-8657 Tokyo, Japan; kaoru.50854@gmail.com (K.T.); akakeiken@g.ecc.u-tokyo.ac.jp (H.J.); takaha_shoko@yahoo.co.jp (S.T.)

**Keywords:** miR-203, protein malnutrition, DNA microarray, miRNA array, hydroxyacyl-CoA dehydrogenase beta subunit, beta-oxidation of fatty acids

## Abstract

Adequate protein nutrition is essential for good health. Effects of protein malnutrition in animals have been widely studied at the mRNA level with the development of DNA microarray technology. Although microRNAs (miRNAs) have attracted attention for their function in regulating gene expression and have been studied in several disciplines, fewer studies have clarified the effects of protein malnutrition on miRNA alterations. The present study aimed to elucidate the relationship between protein malnutrition and miRNAs. Six-week old Wistar male rats were fed a control diet (20% casein) or a low-protein diet (5% casein) for two weeks, and their livers were subjected to both DNA microarray and miRNA array analysis. miR-203 was downregulated and its putative target *Hadhb* (hydroxyacyl-CoA dehydrogenase β subunit), known to regulate β-oxidation of fatty acids, was upregulated by the low-protein diet. In an in vitro experiment, miR-203 or its inhibitor were transfected in HepG2 cells, and the pattern of *Hadhb* expression was opposite to that of miR-203 expression. In addition, to clarifying the hepatic miRNA profile in response to protein malnutrition, these results showed that a low-protein diet increased *Hadhb* expression through downregulation of miR-203 and induced β-oxidation of fatty acids.

## 1. Introduction

As one of the three major nutrients, protein is essential for our lives, and adequate amounts and quality of protein intake are required to maintain good health. It is well known that both excessive and insufficient protein intake lead to several changes in biological functions. For example, excessive protein intake causes a decrease in body weight [1] and influences calcium metabolism [2], while protein malnutrition inhibits growth, causes lipid accumulation in the liver, and leads to protein hypermetabolism [3,4]. Nonetheless, more research on protein nutrition is needed to elucidate its specific contribution to the maintenance of good health in further detail.

In molecular nutritional science, ‘omics’ analyses, also called nutrigenomics, have lately attracted considerable attention from researchers worldwide. The use of one type of omics analyses, transcriptomics, has rapidly proliferated owing to the development of DNA microarrays, which can detect the expression of large numbers of genes and is widely used in the discipline of nutritional research. Using these technologies, protein malnutrition has been shown to regulate the expression of many mRNAs. For example, *Igf1* was found to be downregulated and *Igfbp1* was found to be upregulated in rats fed a low-protein diet [5,6], which is considered to promote protein catabolism.

microRNAs (miRNAs) are small non-coding RNAs that were first reported in 1993 [7,8]. miRNAs regulate gene expression by binding to the 3′UTR of target mRNAs. Regulation of miRNAs is known to have two mechanisms: mRNA cleavage, and transcriptional repression [9,10]. Although the mechanisms remain unclear, changes in mRNA levels closely reflect the impact of miRNAs on gene expression [11].

Several studies on miRNA functions have suggested that miRNAs are involved in various diseases, including cancer, proliferation, invasion, and apoptosis [12,13,14,15,16,17]. The functions of miRNAs have been gradually clarified by these reports; miR-34 induces apoptosis or cell-cycle arrest, and miR-124, miR-24, and miR-629 are related to the inflammatory feedback circuit that regulates hepatocellular oncogenesis. Furthermore, miRNAs have been reported to regulate glucose and lipid metabolism [18,19,20,21,22,23]; miR-103 and miR-107 regulate insulin sensitivity, miR-33a/b regulates fatty acid metabolism and cellular cholesterol efflux, and miR-519d induces lipid accumulation. Thus, miRNA analysis is an important subject, but one that has been rarely been investigated in nutritional science. There have been several reports on the relationship between miRNA expression and maternal or postnatal protein malnutrition [24,25,26], with quite few study in young animals.

The present study focused on protein malnutrition and investigated the relationship between protein nutrition and miRNAs in young rats.

## 2. Materials and Methods

### 2.1. Animals

Male Wistar rats at 6 weeks of age were purchased from Charles River Japan (Kanagawa, Japan) and kept in a room maintained at 22 ± 1 °C with a relative humidity of 60% and a 12-h light–dark cycle (lights on for 8:00–20:00). They were allowed free access to water and food throughout the experiment. After a pre-feeding period of 3 days with the MF diet (Oriental Yeast Co., Ltd., Tokyo, Japan), they were divided into two groups (n = 5) and fed experimental diets for 14 days. The control (C) group was fed a 20% casein diet (AIN-93G), and the low-protein (LP) group was fed a 5% casein diet ad libitum; diet composition is shown in Table 1. During the experimental period, body weight and food intake were measured daily. On day 14, the mice were euthanized and dissected without fasting. After weighing, the livers were frozen and stored at −80 °C. Plasma was obtained by centrifuging the blood at 1000× *g* for 15 min at 4 °C. 

### 2.2. Biochemical Tests

Total lipids in the liver were extracted using the Folch method. Triglyceride (TG), free fatty acid (FFA), and total cholesterol (TC) levels in total lipids in the liver and plasma were measured using the Triglyceride E Test Wako, NEFA C Test Wako, and Cholesterol C Test Wako (Wako Pure Chemical Industries, Ltd., Osaka, Japan), according to the manufacturer’s protocol.

### 2.3. RNA Extraction

Total RNA was extracted from livers using TRIzol reagent (Invitrogen, Carlsbad, CA, USA). The quantity of RNA was measured using a Nanodrop 1000 (Thermo Fisher Scientific, Wilmington, DE, USA), and the quality of total RNA (RIN values >8) was evaluated using an RNA 6000 Nano Assay (Agilent Technologies, Folsom, CA, USA). The quantity of miRNA was measured using a Small RNA Assay (Agilent Technologies, Folsom, CA, USA), which confirmed miRNAs were not lost during the extraction. The experiments were performed according to the manufacturer’s instructions.

### 2.4. miRNA Microarray

The same amounts of RNA from different animals in each group were pooled (n = 5) as one microarray sample and used for poly A tailing and ligation with the FlashTag Biotin HSR RNA Labeling Kit (Affymetrix, Santa Clara, CA, USA). The labeled samples were mixed with a hybridization cocktail (2×Hybridization Mix, 27.5% formamide, DMSO, 20× Eukaryotic Hybridization Controls, Control Oligonucleotide B2 from GeneChip Eukaryotic Hyb Control Kit, Affymetrix) and hybridized onto an miRNA 2.0 Array (Affymetrix) at 48 °C for 16 h. Thereafter, washing and staining were performed using an Affymetrix GeneChip Fluidics Station 450. Scans were performed using an Affymetrix GeneChip Scanner 3000. Data analysis was performed using the Affymetrix miRNA Array QC tool. Each experiment was performed following the manufacturer’s instructions. 

### 2.5. DNA Microarray

Pooled RNA samples (100 ng, n = 5) were amplified and labeled to produce a labeled aRNA using the Affymetrix GeneChip 3′ IVT Express Kit (Affymetrix). Labeled aRNAs were fragmented and hybridized onto a GeneChip Rat Genome 230 2.0 Array (Affymetrix) at 45 °C for 16 h. Washing, staining, and scanning were performed with the same materials as in the miRNA microarray. Data analysis was performed using GeneChip Command Console Software and GeneChip Operating Software (Affymetrix). Each experiment was performed following the manufacturer’s instructions. 

### 2.6. Ingenuity Pathway Analysis (IPA)

The IPA software (Ingenuity Systems, http://www.ingenuity.com/, accessed on 6 January 2013) was used to search for miRNA target genes. Both miRNA microarray data and DNA microarray data were uploaded to IPA, and further analyses were performed using probe sets selected according to the following criteria: relative log ratio value of miRNA expression signal (LP/C) was over 0.5 or under −0.5, detection of miRNAs in either LP group or C group was judged as TRUE, relative log ratio of putative target gene signal was over 1.0 or under −1.0, and detection of mRNA in either the LP group or the C group was judged as present. Subsequently, 6 differentially expressed miRNAs and 2921 differentially expressed genes were applied to the miRNA target filter function of IPA to identify the miRNA target genes.

### 2.7. miRNA Reverse Transcription Polymerase Chain Reaction (RT-PCR)

cDNA was synthesized from total RNA using a miRNA-specific stem–loop RT primer from TaqMan MicroRNA Assays and reagents from the TaqMan microRNA Reverse Transcription Kit (Thermo Fisher Scientific, Wilmington, DE, USA). PCR products were amplified from cDNA using TaqMan MicroRNA Assays with TaqMan Universal PCR Master Mix II, No UNG (Thermo Fisher Scientific). Fluorescence was detected with a Thermal Cycler Dice Real Time System (Takara Bio, Madison, WI, USA). U6 snRNA was used as an internal control.

### 2.8. mRNA RT-PCR

cDNA was synthesized from total RNA using the designed primers and PrimeScript RT Master Mix (Perfect Real Time, Takara Bio). The sequences of the primers were as follows:

*Gapdh* Forward primer: 5′-GGCACAGTCAAGGCTGAGAATG-3′

*Gapdh* Reverse primer: 5′-ATGGTGGTGAAGACGCCAGTA-3′

*Hadhb* Forward primer: 5′-GGAGCAAATGGCCAAACTAA-3′

*Hadhb* Reverse primer: 5′-TTATAACCCATGGCCAGAGC-3′

PCR products were amplified from cDNA using SYBR Premix Ex Taq (Tli RNaseH Plus, Takara Bio). Fluorescence was detected with a Thermal Cycler Dice Real Time System (Takara Bio). *GAPDH* was used as an internal control.

### 2.9. Cell Culture

Human hepatocellular carcinoma cells (HepG2, American Type Culture Collection, Rockville, MD, USA) were cultured in D5796-Dulbecco’s Modified Eagle Medium (D-MEM, Sigma-Aldrich, St. Louis, MO, USA) supplemented with 10% fetal bovine serum (FBS) and 1% penicillin–streptomycin (Sigma-Aldrich). The cells were seeded the day before the experiments to reach 80–90% confluence at the time of transfection. 

### 2.10. Luciferase Assay

HepG2 cells were seeded in 96-well white and clear (to confirm the density of cells) TC plates in a total volume of 100 μL. The synthetic miRNA Target GoClone reporter hsa-miR-203 (SwitchGear Genomics, CA, USA) was used as a target vector, and EMPTY_3UTR, Empty vector (no 3′UTR) (SwitchGear Genomics) was used as a control vector. miCENTURY OX miNatural, has-miR-203 (Cosmo Bio, Tokyo, Japan) was used as the synthetic miR-203. miCENTURY OX miNatural, microRNA control, non-target RNA (Cosmo Bio) was used as the control. miNatural and miRCURY LNA microRNA Inhibitor, hsa-miR-203 (Exiqon, Vedbaek, Denmark) was used as an inhibitor of miR-203. miNatural and miRCURY LNA microRNA inhibitor, antisense control A (Exiqon) was used as the control. Individual vectors and microRNAs were mixed with serum-free media, and Lipofectamine 2000 reagent (Thermo Fisher Scientific) was diluted with Opti-MEM I Reduced Serum Medium (Thermo Fisher Scientific). The final concentrations of synthetic miR-203 controls were 0, 5, 10, 20, and 40 nM, and those of the inhibitor of miR-203 was 40 and 80 nM. The concentration of each control was 40 nM. One hundred microliters of the mixture were added to each well and incubated for 24 h. Luciferase activity was measured using LightSwitch Assay Reagents (SwitchGear Genomics) and Luminescencer JNR IKB2300 (ATTO, Tokyo, Japan) according to the manufacturer’s protocol. 

### 2.11. miRNA Overexpression and Knockdown

HepG2 cells were seeded in 12-well clear TC plates in a total volume of 1 mL. Synthetic miRNA and its inhibitor were the same as those used in the luciferase assay experiment and were diluted in DMEM supplemented with 1% penicillin–streptomycin; the final concentrations were 40 and 80 nM, respectively. Lipofectamine 2000 (Thermo Fisher Scientific) was diluted in Opti-MEM I Reduced Serum Medium (Thermo Fisher Scientific, 1:50 ratio) and incubated for 5 min at room temperature. Diluted synthetic miRNA, its inhibitor, and Lipofectamine 2000 were mixed and incubated for 20 min. The mixture was added to each well and incubated for 24 h. RNA extraction using TRIzol reagent was performed after washing with PBS.

### 2.12. Statistical Analyses 

Data are presented as mean ± standard deviation (SD) and analyzed using one-way analysis of variance. Significant differences were evaluated using Student’s *t*-test and Tukey’s *t*-test at the following levels of significance: * *p* < 0.05 and ** *p* < 0.01.

## 3. Results

### 3.1. LP Diet Affected Lipid Metabolism

The biochemical characteristics of the rats are presented in Table 2. Body weight significantly decreased in the LP group compared to that in the C group. Although there were no significant differences in food intake between the two groups, growth retardation in the LP group was observed throughout the experimental period. Compared to levels in the C group, hepatic TG, NEFA, and TC levels increased, plasma NEFA and HDL-C levels decreased, and plasma TG and TC tended to decrease in the LP group. 

### 3.2. LP Diet Regulated miR-203 and Hadhb mRNA Expression in the Rat Liver 

**Figure 1:**
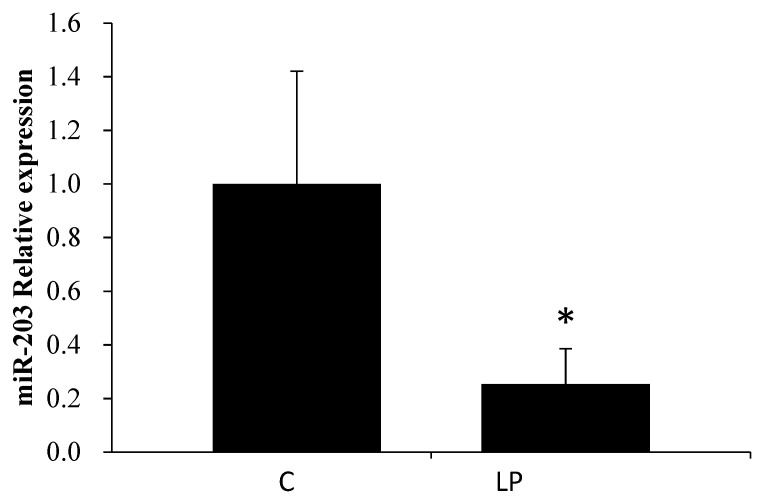
Effect of low-protein diet on miR-203 expression in rat livers measured using RT-PCR. C and LP represent the control and low-protein diet groups, respectively. Values are means ± S.D. (n = 5), * *p* < 0.05 (Student’s *t*-test, vs. C).

**Figure 2:**
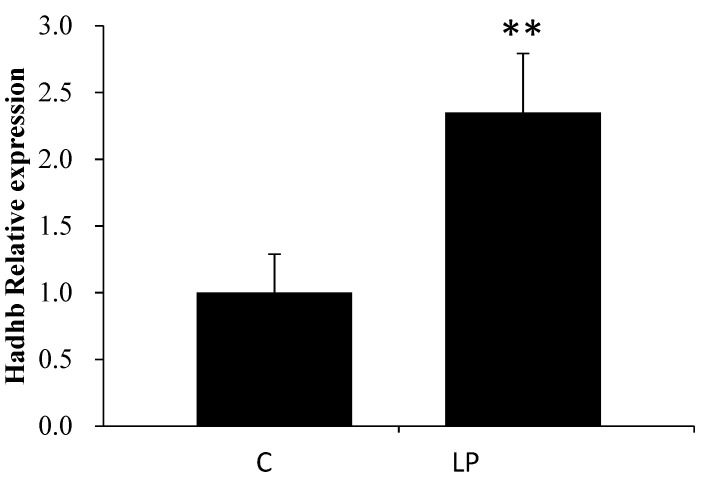
Effect of low-protein diet on mRNA expression of *Hadhb* in rat livers measured using RT-PCR. C and LP represent the control and low-protein diet groups, respectively. Values are means ± S.D. (n = 5), ** *p* < 0.01 (Student’s *t*-test, vs. C).

As shown in Table 3, according to the results of the miRNA microarray, six miRNAs among the detected 389 mature rat miRNAs were differentially expressed in livers of the LP group compared with livers of the C group. The putative target genes of the six miRNAs were searched using IPA software; IPA predicted mRNAs with complementary sequences in the 3′UTR to be putative miRNA target genes. Since miRNA has been reported to repress gene expression, the expression pattern ought to be opposite between miRNAs and their target genes. IPA filtering was based on data from both miRNA and mRNA microarrays (2921 differentially expressed genes); using this function, the putative target genes that showed alterations opposite to those of miRNAs were extracted (Table 4). Since the biochemical characteristics showed LP diet-related alterations in lipid metabolism, we focused on miR-203, whose putative target gene hydroxyacyl-CoA dehydrogenase β subunit (*Hadhb*) is related to lipid metabolism. To confirm the microarray results, the expression of miR-203 and *Hadhb* was measured by RT-PCR (Figure 1 and Figure 2). miR-203 was downregulated and *Hadhb* was upregulated in the liver of the LP group compared to that in the C group, consistent with the microarray results.

### 3.3. miR-203 Regulated the mRNA Expression of Hadhb 

**Figure 3:**
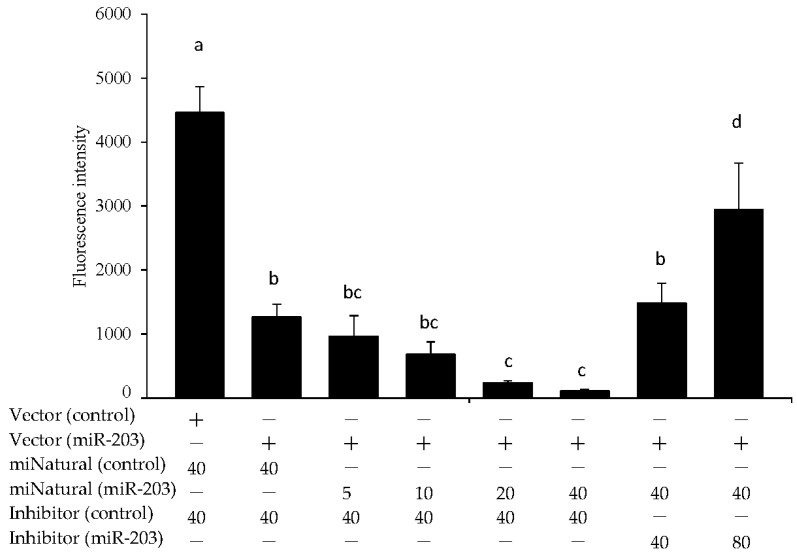
Effect of transfection on expression levels of miR-203 in HepG2 cells, as evaluated by the luciferase assay. Synthetic miR-203 and miR-203 inhibitor were transfected into HepG2 cells in 96-well plates for 24 h. + indicates added, and − indicates not added. The number describes the concentration (nM). Values are means ± S.D (n = 3). Data with different letters (a–c) indicate significant differences (Tukey’s *t*-test, *p* < 0.05).

**Figure 4:**
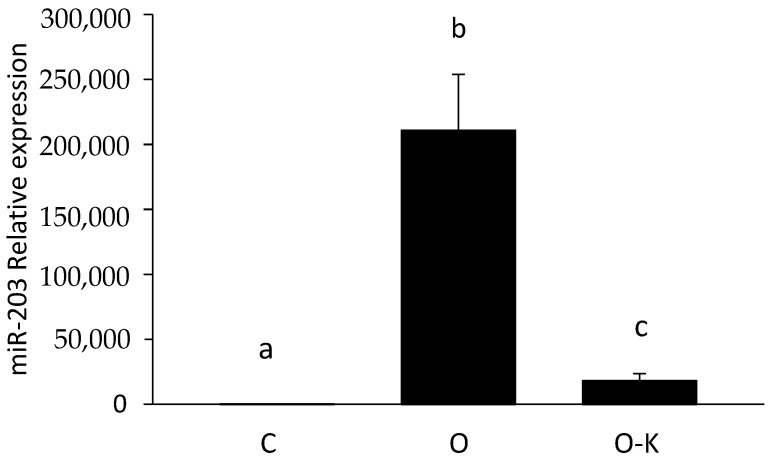
Effect of overexpression and knockdown of miR-203 on the expression of miR-203 in HepG2 cells evaluated by RT-PCR. C, O, and O–K represent control, overexpression, and overexpression–knockdown groups, respectively. Values are means ± S.D. (n = 3). Data with different letters (a–c) indicate significant differences (Tukey’s *t*-test, *p* < 0.01).

**Figure 5:**
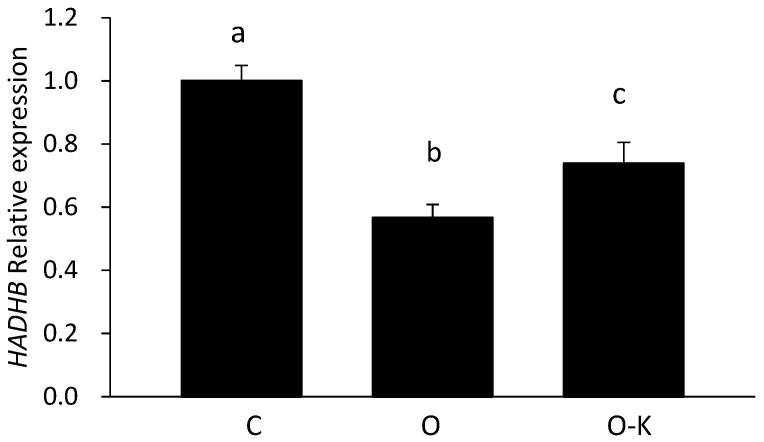
Effect of overexpression and knockdown of miR-203 on mRNA expression of *Hadhb* in HepG2 cells measured by RT-PCR. C, O, and O–K represent control, overexpression, and overexpression–knockdown groups, respectively. Values are means ± S.D. (n = 3). Data with different letters (a–c) indicate significant differences (Tukey’s *t*-test, *p* < 0.01).

To confirm whether miR-203 repressed the mRNA expression of *Hadhb*, synthetic miR-203 and miR-203 inhibitors were transfected into HepG2 cells, and the mRNA expression of *Hadhb* was measured. First, a luciferase assay was performed to measure the expression level of miRNA and determine the concentration of synthetic miR-203 and miR-203 inhibitor (Figure 3). As an miR-203 overexpression experiment, synthetic miR-203 was transfected at 0, 5, 10, 20, and 40 nM. To examine the effect of miR-203 knockdown, miR-203 inhibitor was transfected with synthetic miR-203 because few endogenous miR-203 exist in HepG2 cells. The 40 nM synthetic miR-203 seemed to be adequate to decrease the fluorescent intensity of vectors, and the 80 nM miR-203 inhibitor was adequate to recover this fluorescence intensity. According to these results, 40 nM synthetic miR-203 and 80 nM miR-203 inhibitor were transfected into HepG2 cells. To confirm whether transfection was successful, miR-203 expression was measured by RT-PCR; miR-203 was upregulated in the overexpression (O) group compared with that in the control (C) group and down-regulated in the overexpression and knockdown (O–K) group compared with that in the O group (Figure 4). *Hadhb* expression in transfected HepG2 cells was measured using RT-PCR; *Hadhb* was downregulated in the O group compared with that in the C group and upregulated in the O–K group compared with that in the O group (Figure 5). The expression pattern of *Hadhb* was opposite to that of miR-203.

## 4. Discussion

Here, we investigated for the first time the effect of a low-protein diet on the miRNA profile of rat livers. The presence of several differentially expressed miRNAs in low-protein conditions underscores the importance of the relationship between protein nutrition and miRNAs.

It has been reported that dietary components affect biological functions through the modulation of miRNA expression. For example, retinoid acid induces the differentiation of human acute promyelocytic leukemia through the function of miRNA [27], and curcumin alters miRNA expression profiles in human pancreatic cancer cells [28]. In this study, we demonstrated that protein nutrition also regulates biological phenomena through the function of miRNAs. Our biochemical tests revealed alterations in lipid components (Table 2) of the liver and plasma of LP diet-fed rats. These results confirmed the effect of a low-protein diet on lipid metabolism, as reported previously [3,29,30].

Next, we focused on the relationship between miRNAs and lipid metabolism. To search for responsive miRNAs and their target genes involved in protein malnutrition, two types of microarrays were performed. Although combining two omics datasets is fraught with complications, we compared the results of two microarrays and listed the differentially expressed miRNAs and their predicted target mRNAs simultaneously using IPA. This method enabled us to identify the true target genes and the biological mechanisms regulated by miRNAs. In the present study, we focused on genes related to lipid metabolism because a low-protein diet is known to affect lipid metabolism and is considered to be related to the function of miRNAs. Base on the results of our combined microarray analyses, we focused on miR-203, whose putative target gene *Hadhb* is related to lipid metabolism. Although the *Vldlr* gene is related to the uptake of triglyceride-derived fatty acids [31], the low protein diets induced upregulation of expression of *Hadhb* in rat liver was also confirmed by our previous microarray data obtained from analyses of 5% vs. 15% casein and 6% vs. 12% casein diets. *Hadhb* encodes the β subunit of the mitochondrial trifunctional protein, which catalyzes the last three steps of mitochondrial β-oxidation of long-chain fatty acids, and in the absence of this subunit, the enzyme loses its function [32]. In addition, *Hadhb* is the target gene predicted based on TargetScan data, with a complementary sequence for miR-203 in its 3′ UTR. In the present study, the upregulated expression of *Hadhb* in the rat livers of the LP group suggests that *Hadhb* might be one of the target genes of miR-203. In in vitro experiments, the overexpression of miR-203 downregulated the mRNA expression level of *Hadhb*, and the knockdown of miR-203 reversed this effect (Figure 4 and Figure 5). These results strongly suggest that miR-203 regulates *Hadhb* mRNA expression.

miR-203 is also known to be a tumor-suppressive miRNA in various cancers [14,15,16,17]. In malignant melanoma cells, miR-203 was downregulated and the decreased function of miR-203 was considered to repress cell proliferation [33]. miR-203 also represses cell proliferation in human esophageal squamous cell carcinoma, hematological malignancies, prostate cancer cell lines, and pancreatic adenocarcinoma [34,35,36,37,38]. In hepatocellular carcinoma, miR-203 is also downregulated; its target genes are upregulated and promote tumor formation [31]. The present study demonstrated that miR-203 might also be related to alterations in lipid metabolism caused by protein malnutrition. The accumulation of FFA is known to be toxic, and it accelerates the β-oxidation of fatty acids to reduce toxicity. In this and previous animal experiments, FFA was found to be accumulated in rat livers of the LP group; thus, the β-oxidation of fatty acids was likely to occur, and the up-regulation of *Hadhb* is thought to be one of the causes underlying this mechanism. 

Further studies are required for in-depth investigation of the other candidate miRNAs and genes, as well as to clarify the biological regulatory mechanisms of miR-203 and *Hadhb*, by validating the direct binding of miR-203 to the *Hadhb* untranslated region, confirming the regulation of HADHB at the protein level and the enzyme activity, and examining the effects of miR-203 on lipid metabolism. Nevertheless, we hope that our findings will be beneficial in demonstrating the importance and potential of miRNA analysis in future nutritional, especially protein malnutrition research. 

In conclusion, the findings of this study suggest that protein nutrition regulates biological phenomena through the function of miRNAs. The present study clarified the hepatic miRNA profile in response to protein malnutrition and showed that a low-protein diet increased the expression of *Hadhb* through the downregulation of miR-203 and induced β-oxidation of fatty acids.

## Figures and Tables

**Table 1:** Diet composition.

	20% Casein Diet (AIN-93G)	5% Casein Diet
Cornstarch (g)	397.5	547.5
Casein (g)	200	50
Dextrinized cornstarch (g)	132	132
Sucrose (g)	102.5	102.5
Soybean oil (g)	70	70
Fiber (g)	50	50
Mineral mix (g)	35	35
Vitamin mix (g)	10	10
L-cysteine (g)	3	3
Total (g)	1000	1000

**Table 2:** Biochemical parameters.

	C	LP
Final body weight (g)	341.3 ± 22.2	289.2 ± 12.9 **
Liver weight (g/BW)	4.3 ± 0.2	4.7 ± 0.4
TG (mg/g tissue)	11.3 ± 3.1	59.4 ± 13.0 **
TC (mg/g tissue)	4.5 ± 0.3	7.4 ± 1.1 **
FFA (mEq/g tissue)	21.4 ± 2.3	119.4 ± 52.7 **
Plasma TG (mg/dL)	206.0 ± 32.0	178.0 ± 66.2
TC (mg/dL)	63.6 ± 15.0	47.9 ± 6.4
FFA (mEq/L)	0.23 ± 0.02	0.14 ± 0.06 *
HDL-C (mg/dL)	31.0 ± 6.3	21.6 ± 3.2 *

Values are means ± S.D. (n = 5). * *p* < 0.05, ** *p* < 0.01 (Student’s *t*-test, vs. C).

**Table 3:** Results of the miRNA 2.0 array.

miRNA	miRNA-Log Ratio
**upregulated**	
rno-miR-200b	1.1
rno-miR-429	0.5
**downregulated**	
rno-miR-203	−1.1
rno-miR-193	−1.1
rno-miR-182	−0.9
rno-miR-210	−0.6

Six miRNAs were differentially expressed in rat livers of the LP group compared to livers of the C group. The relative log ratio of miRNA expression signal (LP/C) was >0.5 or <−0.5. The detection of upregulated miRNAs in the LP group and downregulated miRNAs in the C group was TRUE.

**Table 4:** Various miRNAs and their putative target genes.

miRNA	Log Ratio	Putative Target Genes	Log Ratio
rno-miR-200b	1.1	*Fhl1* *Runx1t1* *Ccna2, Slc6a6* *Mmd2* *Kiaa0101, Lox* *Mpdz* *CIted2, Gpm6a, Mbnl3, Xkr8* *Ptprd*	−4.6 −3.5 −2.3 −1.9 −1.8 −1.3 −1.2 −1.0
rno-miR-203	−1.1	*Prickle2* *Fkbp5* *Hadhb* *Rpl23a*	4.0 1.3 1.2 1.0
rno-miR-193	−1.1	*Ccnd1* *Agpat3, Atf5*	1.3 1.0
rno-miR-182	−0.9	*Foxq1* *Vldlr* *Trib3* *Shc4* *Glra3* *Tsku* *Lpar4* *Arhgef2, Camkk2, Fam118a, Otub2* *Taf4b* *Npm1* *Tmem86a*	4.9 4.2 3.6 3.2 3.0 1.8 1.7 1.6 1.5 1.1 1.0
rno-miR-210	−0.6	*Slc7a11*	2.1

Differentially expressed miRNAs in the LP group compared with the C group in the miRNA array analysis and each putative target gene are shown. The log ratio of the putative target gene signal was >1.0 or <−1.0. Fhl1, four and a half LIM domains 1; Runx1t1, runt-related transcription factor 1 translocated to 1; Ccna2, cyclin A2; Slc6a6, solute carrier family 6, member 6; Mmd2, monocyte to macrophage differentiation-associated 2; Lox, lysyl oxidase; Mpdz, multiple PDZ domain protein; Cited2, Cbp/p300-interacting transactivator, with Glu/Asp-rich carboxy-terminal domain, 2; Gpm6a, glycoprotein m6a; Mbnl3, muscleblind-like 3; Xkr8xk, Kell blood group complex subunit-related family, member 8; Ptprd, protein tyrosine phosphatase, receptor type, D; Prickle2, prickle homolog 2; Fkbp5, FK506 binding protein 5; Hadhb, hydroxyacyl-CoA dehydrogenase β subunit; Rpl23a, ribosomal protein L23a; Ccnd1, cyclin D1; Agpat3, 1-acylglycerol-3-phosphate O-acyltransferase 3; Atf5, activating transcription factor 5; Foxq1, Forkhead box Q1; Vldlr, very low density lipoprotein receptor; Trib3, tribbles homolog 3; Shc4, SHC family, member 4; Glra3, glycine receptor, α 3; Tsku, tsukushin; Lpar4, lysophosphatidic acid receptor 4; Arhgef2, rho/rac guanine nucleotide exchange factor 2; Camkk2, calcium/calmodulin-dependent protein kinase kinase 2, β; Fam118a, family with sequence similarity 118, member A; Otub2otu, domain, ubiquitin aldehyde binding 2; Taf4b, TAF4B RNA polymerase II, TATA box binding protein-associated factor; Npm1, nucleophosmin; Tmem86a, transmembrane protein 86A; Slc7a11, solute carrier family 7, member 11.

## Data Availability

The data presented in this study are available on request from the corresponding author.
